# Protective Effects of Ginseng Extract Against Oxidative Stress in Chilled Rooster Semen: Implications for Sperm Quality and Fertility

**DOI:** 10.3390/ani16131960

**Published:** 2026-06-25

**Authors:** Ruthaiporn Ratchamak, Khanitta Pengmeesri, Eakapol Wangkahart

**Affiliations:** 1 Division of Animal Science, Department of Agricultural Technology, Faculty of Technology, Mahasarakham University, Maha Sarakham 44150, Thailand; khanitta.c@msu.ac.th; 2 Division of Fisheries, Department of Agricultural Technology, Faculty of Technology, Mahasarakham University, Maha Sarakham 44150, Thailand; eakapol.w@msu.ac.th; 3 Laboratory of Fish Immunology and Nutrigenomics, Applied Animal and Aquatic Sciences Research Unit, Division of Fisheries, Faculty of Technology, Mahasarakham University, Maha Sarakham 44150, Thailand

**Keywords:** antioxidant, ginseng, lipid peroxidation, oxidative stress

## Abstract

Oxidative stress is a primary driver of sperm deterioration during chilled storage of poultry semen, and identifying effective natural antioxidant supplements for semen extenders is an important practical goal for poultry reproductive management. This study evaluated the protective effects of ginseng extract (Panax ginseng) supplementation on sperm viability, motility, oxidative stress biomarkers, antioxidant defense, and fertility in chilled Leung Hang Kao rooster semen. Pooled semen was diluted in IGGKPh extender supplemented with ginseng extract at 0, 1, 2, 3, or 4 mg/mL and stored at 5 °C for 0, 24, and 48 h. Sperm viability, total motility, progressive motility, malondialdehyde (MDA) concentration, total antioxidant capacity (T-AOC), glutathione peroxidase (GPx) activity, catalase (CAT) activity, and fertility following artificial insemination were evaluated at each time point. All ginseng-supplemented groups showed significantly lower MDA concentrations and higher GPx activity than the unsupplemented control throughout storage. At 48 h, total motility and progressive motility were highest in the 2 and 3 mg/mL groups, while T-AOC was best maintained in the 1 and 2 mg/mL groups. CAT activity did not differ significantly among groups at 48 h (*p* = 0.2498). Fertility was significantly higher in the 1 and 2 mg/mL groups than in the control after 24 and 48 h of storage, and the alignment between T-AOC and fertility across storage time points indicated that overall antioxidant buffering capacity was a stronger determinant of fertilizing competence than individual enzyme activities or MDA concentration alone. Concentrations of 3–4 mg/mL, despite producing lower MDA at 48 h, did not confer superior fertility outcomes, suggesting a hormetic dose–response relationship. Based on integrated evidence from sperm quality, antioxidant status, and in vivo fertility, ginseng extract supplementation at 1–2 mg/mL is recommended as the most suitable range for preserving chilled Leung Hang Kao rooster semen and may represent a practical natural antioxidant strategy for Thai native poultry breeding programs.

## Simple Summary

Short-term chilled storage of rooster semen is a practical and widely used approach in poultry breeding programs, but sperm quality commonly declines during storage owing to the high susceptibility of avian spermatozoa to oxidative damage. Identifying safe, natural additives that protect sperm function during liquid storage could meaningfully improve semen preservation outcomes and support reproductive efficiency in poultry. Ginseng (Panax ginseng) is a well-recognized medicinal plant whose bioactive constituents, particularly ginsenosides, possess potent antioxidant and cytoprotective properties that have been demonstrated across multiple biological systems. In this study, we examined whether supplementing a semen extender with ginseng extract at graded concentrations could protect chilled semen from Leung Hang Kao roosters, a Thai native breed, during storage at 5 °C for up to 48 h. We assessed sperm viability, motility, lipid peroxidation, total antioxidant capacity, antioxidant enzyme activities, and fertility following artificial insemination. Our results showed that ginseng extract at 1–2 mg/mL most effectively preserved sperm function and fertilizing capacity during storage, whereas higher concentrations provided less consistent benefit. These findings suggest that ginseng extract is a promising natural supplement for chilled rooster semen extenders and may contribute to the development of practical, plant-based strategies for improving semen handling and fertility outcomes in Thai native poultry breeding programs.

## 1. Introduction

Artificial insemination (AI) using liquid-stored semen is an important reproductive technology in both turkey and native chicken production. In turkeys, AI is necessitated primarily by the physical incompatibility between males and females arising from intensive selection for large body size, which renders natural mating difficult or impossible [1]. In Thai native chickens, AI is increasingly applied in semi-intensive and conservation breeding systems to increase the effective mating ratio and maximize the reproductive contribution of genetically valuable males [2,3]. However, the fertilizing capacity of rooster semen is highly time-sensitive; undiluted raw rooster semen undergoes rapid, progressive deterioration in motility and fertilizing capacity within 1 h of collection [4], making immediate dilution in an appropriate extender essential for maintaining sperm viability during short-term liquid storage.

Despite the protective role of extenders in stabilizing pH and osmolarity and in providing metabolic substrates, sperm quality declines progressively during chilled storage due to the continuous accumulation of reactive oxygen species (ROS), lipid peroxidation of plasma membrane phospholipids, and oxidative damage to mitochondrial and nuclear components, which collectively impair sperm motility, viability, and fertilizing capacity [5,6]. Avian spermatozoa are particularly vulnerable to oxidative damage because their plasma membranes contain exceptionally high concentrations of long-chain polyunsaturated fatty acids (PUFAs), which serve as primary substrates for ROS-initiated chain reactions of lipid peroxidation [7,8,9]. Moreover, avian spermatozoa possess relatively limited cytoplasmic volume and consequently harbor sparse endogenous antioxidant defenses, rendering them dependent on the extracellular antioxidant environment provided by the seminal plasma and, during ex vivo storage, by the semen extender [10,11].

These biological characteristics have motivated growing research interest in optimizing extender composition and antioxidant supplementation to preserve Thai native rooster semen. Studies comparing extender formulations have demonstrated that EK and IGGKPh extenders better preserved sperm motility and viability than NaCl-based diluents, with concomitantly lower malondialdehyde (MDA) concentration and ROS accumulation during chilled storage [6], while the NCAB extender enhanced sperm viability and fertilizing ability relative to saline and IGGKPh [2]. Antioxidant supplementation has further improved preservation outcomes; phosphorus and vitamin B12 supplementation of chilled rooster semen preserved motility and reduced oxidative stress markers [12] and Kaempferia parviflora extract maintained sperm viability and fertility during liquid storage [13]. Collectively, these findings establish oxidative stress suppression and plasma membrane stabilization as the central determinants of successful short-term liquid preservation in Thai native roosters and highlight the potential of plant-derived antioxidants as practical, accessible supplements for semen extenders in this context.

Panax ginseng contains a diverse array of triterpene saponins, collectively termed ginsenosides, which protect spermatozoa against oxidative damage through multiple complementary mechanisms, including direct ROS scavenging, attenuation of lipid peroxidation, upregulation of endogenous antioxidant enzymes including superoxide dismutase (SOD), catalase (CAT), and glutathione peroxidase (GPx), enhancement of total antioxidant capacity (T-AOC), and preservation of mitochondrial membrane integrity [14,15]. In poultry, dietary ginseng supplementation has been associated with improvements in semen quality parameters, and its inclusion in rooster cryopreservation media significantly reduced post-thaw lipid peroxidation, enhanced antioxidant enzyme activities, and improved fertility [16,17]. Supporting evidence from mammalian liquid storage models further demonstrates that ginseng-derived compounds can preserve sperm motility, membrane integrity, and antioxidant defense under metabolically active, non-frozen storage conditions [18], suggesting that their protective mechanisms are relevant across both cryopreservation and chilled storage paradigms.

Nevertheless, the efficacy of ginseng extract as a supplement for chilled semen extender remains uninvestigated in Thai native roosters. Unlike cryopreservation, chilled storage maintains spermatozoa in a metabolically active state at reduced but non-zero metabolic rates, allowing oxidative damage to accumulate continuously throughout the storage period; therefore, findings from freezing studies cannot be directly extrapolated to liquid preservation. Furthermore, the dose–response relationship between ginseng extract concentration and chilled semen preservation outcomes has not been characterized in any avian species, and the possibility of a hormetic response—in which moderate concentrations confer protection, whereas supraoptimal concentrations produce cytotoxic or prooxidant effects—has not been addressed. The present study, therefore, graded concentrations of standardized Panax ginseng extract (0, 1, 2, 3, and 4 mg/mL) supplemented into the IGGKPh extender for Leung Hang Kao rooster semen, with respect to sperm viability, total and progressive motility, MDA concentration, T-AOC, GPx and CAT activities, and in vivo fertility following artificial insemination after 0, 24, and 48 h of storage at 5 °C. We hypothesized that ginseng extract supplementation would attenuate oxidative stress, improve antioxidant buffering capacity, and help preserve sperm functional integrity and fertilizing ability during chilled storage in a concentration-dependent manner. The graded concentration design was also intended to identify an effective concentration range and to evaluate whether higher concentrations produced diminishing protective effects suggestive of a hormetic dose–response pattern.

## 2. Materials and Methods

### 2.1. Experimental Animals and Chemicals

All experimental procedures were reviewed and approved by the Institutional Animal Care and Use Committee, Mahasarakham University, Thailand [IACUC-MSU-42/2024]. Thirty Leung Hang Kao roosters, aged 48 weeks, were individually housed in battery cages under a controlled photoperiod of 16 h light: 8 h dark and provided approximately 120 g of commercial broiler breeder feed per day with ad libitum access to fresh water.

For the fertility trial, thirty commercial hens aged 32 weeks, with a laying rate exceeding 95%, were individually housed in laying cages and had no prior contact with males. Hens were provided approximately 110 g of commercial layer feed per day with ad libitum access to water throughout the experimental period.

All chemical substances were purchased from Sigma-Aldrich Chemical Company (St. Louis, MO, USA), unless otherwise stated. Chemicals were weighed using a calibrated analytical balance (readability 0.1 mg) and prepared under aseptic conditions. The IGGKPh extender was prepared according to Ratchamak et al. [5] and consisted of 0.14 g potassium citrate, 1.40 g sodium glutamate, 0.21 g sodium dihydrogen phosphate, 0.98 g disodium hydrogen phosphate, 0.90 g glucose, and 0.90 g inositol dissolved in 100 mL deionized water, yielding a pH of 7.8 and osmolality of 460 mOsm/kg. The extender was sterilized by passage through a 0.22 µm syringe filter and stored at 4 °C overnight prior to use.

Ginseng extract (standardized Panax ginseng root extract; [G7253, Sigma-Aldrich (St. Louis, MO, USA)]) was dissolved in the IGGKPh extender at concentrations of 0, 1, 2, 3, and 4 mg/mL by vortex mixing for 1 min, followed by overnight incubation at 5 °C to ensure complete dissolution. Each preparation was then passed through a sterile 0.22 µm syringe filter to remove particulates prior to use.

### 2.2. Experimental Design

The experiment was conducted using a completely randomized design (CRD) with five treatment groups corresponding to ginseng extract concentrations of 0 (control), 1, 2, 3, and 4 mg/mL in the IGGKPh extender. Diluted semen samples were stored at 5 °C. Sperm quality assessments were performed at 0, 24, and 48 h of storage, encompassing evaluation of sperm motility parameters, viability, lipid peroxidation, and antioxidant enzyme activities. Fertility was assessed through artificial insemination, with outcomes measured as fertility rate and hatchability of fertile eggs. Each treatment was replicated six times, with each replicate representing an independent semen collection from the rooster pool and serving as the experimental unit for statistical analysis.

### 2.3. Semen Collection and Chilled Semen Preservation

Semen was collected twice weekly from each rooster using the dorso-abdominal massage technique [19]. Individual ejaculates were collected directly into 1.5 mL microtubes pre-warmed to 25 °C and containing 100 µL of IGGKPh extender to minimize osmotic shock upon collection. Samples were maintained at 25 °C, protected from direct sunlight, and transported to the laboratory within 20–30 min in a foam-insulated container.

Upon arrival, samples were gently mixed by inverting the tubes three times to ensure homogeneity prior to quality assessment. Fresh semen from individual roosters was assessed for basic quality parameters, and only ejaculates meeting the minimum acceptance criteria of sperm concentration ≥3 × 10^9^ spermatozoa/mL, total motility ≥80%, and sperm viability ≥80% were retained for further use. Qualified ejaculates were pooled to reduce inter-individual variability.

Pooled semen was diluted with the assigned ginseng-supplemented IGGKPh extender at a ratio of 1:3 (*v*/*v*), yielding a final sperm concentration of approximately 150 × 10^6^ spermatozoa/mL. Following dilution, samples were loaded into 0.25 mL French straws, sealed, and placed in a programmable cooling device. Samples were gradually cooled from 25 °C to 5 °C over 60 min to minimize cold shock, and then stored at 5 °C.

### 2.4. Fresh Semen Evaluation

Initial semen quality was assessed according to Chankitisakul et al. [20]. Semen volume was measured using a calibrated 1 mL syringe (Nipro Company Limited, Phra Nakhon Si Ayutthaya, Thailand). Sperm concentration was determined using a hemocytometer (Neubauer improved chamber). Briefly, 5 µL of semen was diluted in 195 µL of 4% (*w*/*v*) sodium chloride solution (1:40 dilution), and a 10 µL aliquot was loaded onto the hemocytometer. Sperm were counted under a compound microscope at 400× magnification, and concentration was expressed as 10^7^ spermatozoa/mL. Sperm motility assessment is described in Section 2.5, and viability assessment is described in Section 2.6.

### 2.5. Sperm Motility Assessment

Sperm motility was evaluated using a computer-assisted sperm analysis (CASA) system (Hamilton Thorne Biosciences, Beverly, MA, USA; IVOS II, version 12 TOX VIOS) coupled with Olympus microscopy software. A 5 µL aliquot of each semen sample was loaded into a pre-warmed analysis chamber (37 °C). The following acquisition settings were applied: frame rate, 60 frames/s; number of frames captured, 30; minimum head brightness, 165; minimum cell size, 2 μm; maximum cell size, 50 μm. A minimum of 300 spermatozoa across at least five microscopic fields was analyzed per sample. Total motility (%) was defined as the percentage of spermatozoa exhibiting any detectable movement, while progressive motility (%) was defined as the percentage of spermatozoa exhibiting predominantly forward, directional movement.

### 2.6. Sperm Viability

Sperm viability was assessed using the eosin-nigrosin staining technique. Briefly, 15 µL of semen was mixed with 30 µL of eosin-nigrosin stain on a clean glass slide, smeared to produce a thin, uniform film, and allowed to air-dry completely. Slides were examined under a light microscope at 1000× magnification. A minimum of 300 spermatozoa per sample were evaluated and classified as live (unstained) or dead (eosin-stained pink). Sperm viability was expressed as the percentage of live spermatozoa relative to the total number evaluated.

### 2.7. Lipid Peroxidation

Lipid peroxidation was quantified by measuring malondialdehyde (MDA) concentration in seminal plasma generated under induced oxidative conditions using the thiobarbituric acid reactive substances (TBARS) assay, as described by Ratchamak et al. [16]. Briefly, 0.25 mL of semen samples was mixed with 0.25 mL of ferrous sulfate (0.2 mM) and 0.25 mL of ascorbic acid (1 mM) and incubated at 37 °C for 60 min in a water bath to initiate lipid peroxidation. Subsequently, 1 mL of trichloroacetic acid [15% (*w*/*v*)] and 1 mL of thiobarbituric acid [0.375% (*w*/*v*)] were added, and the mixture was boiled for 10 min. Samples were immediately cooled to 4 °C to terminate the reaction, and then centrifuged at 800× *g* for 10 min at 4 °C. The absorbance of the resulting supernatant was measured at 532 nm using a UV–Visible spectrophotometer (Analytik Jena, Specord 250 Plus, Jena, Germany). MDA production was calculated using a standard curve of 1,1,3,3-tetramethoxypropane and expressed as µmol MDA equivalents/mL semen according to the referenced TBARS protocol.

### 2.8. Antioxidant Enzyme Activities

Antioxidant enzyme activities in seminal plasma were assessed by spectrophotometric methods following the methodology described by Chankitisakul et al. [20], to evaluate the oxidative defense capacity of chilled semen during storage.

Total antioxidant capacity (T-AOC) was determined using the 2,2-diphenyl-1-picrylhydrazyl (DPPH) radical scavenging assay. Briefly, 20 µL of seminal plasma was mixed with 180 µL of 0.2 mM DPPH solution and incubated at 30 °C for 30 min in darkness. Absorbance was recorded at 520 nm, and T-AOC was expressed as the percentage of DPPH radical inhibition relative to a blank control.

Glutathione peroxidase (GPx) activity was evaluated by monitoring the rate of NADPH oxidation at 340 nm. The reaction mixture contained seminal plasma, reduced glutathione (GSH), glutathione reductase, NADPH, and tert-butyl hydroperoxide as substrate. Sodium azide was added to inhibit endogenous catalase activity. GPx activity was calculated using the molar extinction coefficient of NADPH (6220 M^−1^ cm^−1^) and expressed as U/mL.

Catalase (CAT) activity was determined by measuring the rate of hydrogen peroxide (H_2_O_2_) decomposition at 230 nm. Seminal plasma was mixed with Tris/EDTA buffer and H_2_O_2_ (50 mM), and absorbance was recorded every 5 s over 8 min at 30 °C. CAT activity was calculated using the molar extinction coefficient of H_2_O_2_ (81 M^−1^ cm^−1^) and expressed as U/mL.

### 2.9. Fertility Assessment

The fertilizing capacity of chilled semen from each treatment group was evaluated through AI of laying hens under controlled conditions. A total of 30 hens were used and randomly allocated to five treatment groups, giving six hens per ginseng concentration group. Each hen was inseminated once weekly with a standardized dose of 0.4 mL of extended semen, delivering approximately 400 × 10^6^ spermatozoa per insemination, administered into the everted oviduct using a sterile inseminating pipette. All inseminations were performed between 15:00 and 16:00 h to synchronize with the hen’s ovulatory cycle and maximize sperm residence time in the oviduct. Eggs were collected daily from day 2 post-insemination onward. Fertility was determined by candling on day 7 of incubation and expressed as the percentage of fertile eggs relative to the total number of eggs set.

### 2.10. Statistical Analysis

All data were analyzed using a one-way analysis of variance (ANOVA) under a CRD, with ginseng extract concentration as the sole treatment factor. Prior to analysis, data were examined for normality using the Shapiro–Wilk test and for homogeneity of variances using Levene’s test. Where ANOVA revealed significant treatment effects, pairwise comparisons among treatment means were performed using Tukey’s honest significant difference (HSD) post hoc test. Statistical significance was declared at *p* < 0.05. All results are presented as mean ± standard error (SE). Statistical analyses were performed using SAS software (Version 9.0, SAS Institute Inc., Cary, NC, USA).

## 3. Results

### 3.1. Fresh Semen Characteristics

Fresh semen characteristics of Leung Hang Kao roosters are presented in Table 1. All quality parameters met or exceeded the predefined minimum acceptance criteria, confirming the suitability of the collected ejaculates for inclusion in the chilled semen preservation experiment.

### 3.2. Effects of Ginseng Extract on Sperm Viability and Motility During Chilled Storage

Sperm viability, total motility, and progressive motility were significantly affected by ginseng extract concentration at all storage time points (*p* < 0.05), with all parameters declining progressively across all treatment groups over the 48 h storage period (Table 2).

**Sperm viability** differed significantly among treatment groups at all time points. At 0 h, the 1 mg/mL group exhibited the highest viability (81.41%), which was significantly greater than the control (65.75%; *p* < 0.05), whereas the remaining supplemented groups (2, 3, and 4 mg/mL) were intermediate and did not differ significantly from either the control or the 1 mg/mL group. After 24 h of storage, viability was significantly higher in the 1, 2, and 3 mg/mL groups compared with both the control and 4 mg/mL groups (*p* < 0.05). By 48 h, the 2 and 3 mg/mL groups maintained the highest viability, which was significantly higher than that of the control and 4 mg/mL groups (*p* < 0.05), while both the control and 4 mg/mL groups recorded the lowest values.

**Total motility** was also significantly influenced by ginseng extract supplementation at all storage time points. At 0 h, the 1 mg/mL group exhibited the highest total motility (80.95%), which was significantly greater than the control and 4 mg/mL groups (69.19% and 69.77%, respectively; superscript c), while the 2 and 3 mg/mL groups were intermediate (superscript b; *p* < 0.05). After 24 h, the 1, 2, and 3 mg/mL groups collectively maintained significantly higher total motility than the control and 4 mg/mL groups (*p* < 0.05). By 48 h, the 2 and 3 mg/mL groups recorded the highest total motility (76.60% and 76.95%, respectively), which were significantly greater than the control (61.23%; *p* < 0.05), while the 4 mg/mL group was intermediate and the control remained the lowest.

**Progressive motility** exhibited a pattern consistent with the above parameters. At 0 h, the 1 mg/mL group showed the highest progressive motility (52.27%), which was significantly greater than the 2 and 3 mg/mL groups (intermediate; superscript b) and the control and 4 mg/mL groups, which recorded the lowest values (40.82% and 40.12%, respectively; superscript c; *p* < 0.05). After 24 h, the 2 mg/mL group exhibited the highest progressive motility (49.45%), which was significantly greater than the 4 mg/mL group (39.76%; *p* < 0.05), while the control (40.85%) was also lower than the 2 mg/mL group. By 48 h, the 2 and 3 mg/mL groups maintained the highest progressive motility (43.87% and 44.45%, respectively), which were significantly greater than the control (28.20%; *p* < 0.05), while the 1 and 4 mg/mL groups were intermediate and did not differ significantly from either extreme.

### 3.3. Effects of Ginseng Extract on Lipid Peroxidation and Antioxidant Activities

The effects of ginseng extract supplementation on lipid peroxidation and antioxidant status during chilled storage are presented in Table 3. Malondialdehyde concentration was significantly affected by treatment at all storage time points (*p* < 0.05). At 0 h, all ginseng-supplemented groups exhibited significantly lower MDA concentrations than the control (*p* < 0.05), with no significant differences among supplemented groups. After 24 h, the 1 mg/mL group had the lowest MDA concentration, which was significantly lower than that of the control (*p* < 0.05), while the 2, 3, and 4 mg/mL groups were intermediate. By 48 h, the 3 and 4 mg/mL groups maintained the lowest MDA concentrations, significantly lower than the control (*p* < 0.05), while the 1 and 2 mg/mL groups were intermediate.

Total antioxidant capacity was significantly influenced by ginseng supplementation at all storage time points (*p* < 0.05). At 0 and 24 h, the 1 mg/mL group consistently exhibited the highest T-AOC, significantly exceeding the 3 mg/mL group at both time points (*p* < 0.05), while the control, 2, and 4 mg/mL groups were intermediate. By 48 h, the pattern shifted, with the 1 and 2 mg/mL groups maintaining the highest T-AOC values, which were significantly greater than those of the 4 mg/mL group (*p* < 0.05), while the control and 3 mg/mL groups were intermediate.

Both GPx and CAT activities were significantly affected by ginseng supplementation at 0 and 24 h (*p* < 0.05), with the control consistently recording the lowest values at both time points. At 0 h, the highest activities were recorded in the 4 mg/mL group for GPx (4.58 U/mL) and the 2 mg/mL group for CAT (10.23 U/mL), with the remaining groups intermediate and not significantly different from the control. After 24 h, both enzymes responded consistently, with the 4 mg/mL group recording the highest GPx (6.67 U/mL) and CAT (14.07 U/mL) activities, significantly exceeding the control (3.83 and 8.65 U/mL, respectively; *p* < 0.05); for CAT, the 3 mg/mL group was the next highest (superscript b), followed by the 1 and 2 mg/mL groups (superscript bc), while the control was lowest (superscript c). By 48 h, GPx activity remained significantly higher in all supplemented groups than in the control (*p* < 0.05), with no significant differences among supplemented treatments, whereas CAT activity did not differ significantly among any groups (*p* = 0.2498).

### 3.4. Effects of Ginseng Extract on Fertility

**Figure 1:**
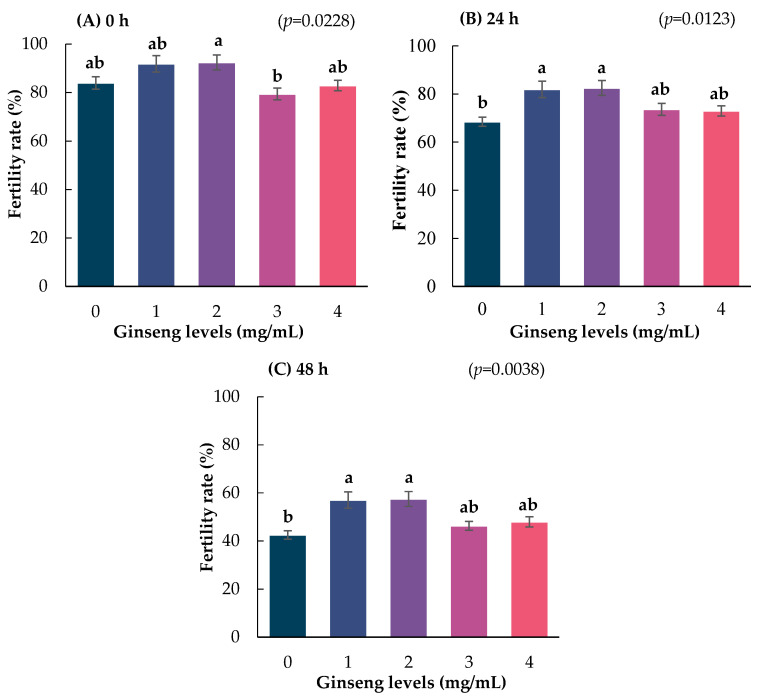
Fertility rates (%) of broiler breeder hens inseminated with rooster semen supplemented with ginseng extract at concentrations of 0, 1, 2, 3, and 4 mg/mL following chilled storage at 5 °C for 0 h (**A**), 24 h (**B**), and 48 h (**C**). Values are presented as mean ± SEM. Bars with different superscript letters differ significantly (*p* < 0.05).

Fertility rates of broiler breeder hens inseminated with chilled rooster semen are presented in Figure 1. Significant differences among treatment groups were observed at all storage time points (*p* < 0.05).

At 0 h of storage, the 2 mg/mL group recorded the highest fertility rate (92.45%), which was significantly greater than the 3 mg/mL group (79.44%; *p* < 0.05). All remaining groups—including the control (83.96%), 1 mg/mL (91.90%), and 4 mg/mL (82.93%)—were intermediate and did not differ significantly from either the 2 or 3 mg/mL groups.

At 24 and 48 h, a consistent pattern emerged: the 1 and 2 mg/mL groups maintained significantly higher fertility rates than the control group at both time points (*p* < 0.05). At 24 h, fertility in the 1 and 2 mg/mL groups (81.90% and 82.45%, respectively) was significantly greater than in the control (68.46%), whereas the 3 and 4 mg/mL groups (73.61% and 72.93%, respectively) were intermediate and did not differ significantly from either the control or the 1–2 mg/mL groups. A similar pattern was maintained at 48 h, with the 1 and 2 mg/mL groups recording the highest fertility rates (57.00% and 57.45%, respectively), significantly exceeding the control (42.46%; *p* < 0.05), while the 3 and 4 mg/mL groups (46.27% and 47.93%, respectively) were again intermediate. Notably, fertility declined progressively across all treatment groups as storage duration increased, yet the significant advantage of 1 and 2 mg/mL supplementation over the control was consistently maintained through 48 h of chilled storage.

## 4. Discussion

The present study demonstrated that ginseng extract supplementation improved the preservation of chilled Leung Hang Kao rooster semen at 5 °C, as evidenced by better maintenance of sperm viability and motility, reduced lipid peroxidation, modulation of antioxidant enzyme activities, and enhanced post-storage fertility. Although benefits were observed across supplemented groups, the pattern of response differed markedly among parameters: fertility and total antioxidant capacity were best maintained at 1–2 mg/mL, whereas GPx and CAT activities were highest at 3–4 mg/mL, and MDA was lowest at 3–4 mg/mL at 48 h. This dissociation between individual biochemical endpoints and reproductive outcomes indicates that the value of ginseng lies not in maximizing any single antioxidant response, but in achieving a concentration range that maintains the overall redox environment compatible with sperm functional integrity. The central question raised by these findings is therefore not simply whether ginseng acts as an antioxidant, but which aspects of oxidative stress modulation are most mechanistically relevant to preserving fertilizing competence during chilled storage.

The reduction in MDA concentration across ginseng-supplemented groups provides the clearest mechanistic basis for this interpretation. Avian spermatozoa are inherently susceptible to oxidative injury owing to the exceptionally high proportion of polyunsaturated fatty acids in their plasma membranes, which serve as preferential substrates for lipid peroxidation during in vitro preservation [7,8]. Under chilled conditions, spermatozoa remain metabolically active, sustaining basal mitochondrial respiration, ion regulation, and flagellar activity, all of which continuously generate ROS [5,21,22]. Once ROS production exceeds the antioxidant buffering capacity of the storage system, oxidative damage accumulates progressively in the sperm membrane and flagellar apparatus, leading to loss of membrane integrity, impaired motility, and reduced fertilizing ability [23,24]. In the present study, MDA concentrations declined across all supplemented groups relative to the control at early storage, consistent with ginseng-mediated suppression of early lipid peroxidation. However, by 48 h, the lowest MDA values were observed in the 3 and 4 mg/mL groups rather than the 1–2 mg/mL groups, yet fertility was significantly higher in the latter. This dissociation suggests that MDA concentration alone does not fully account for the observed fertility differences, and that additional mechanisms related to overall antioxidant buffering capacity are likely involved.

In this regard, the pattern of total antioxidant capacity provides a more coherent explanation of the fertility outcomes. T-AOC was consistently highest in the 1–2 mg/mL groups at both 24 and 48 h of storage, mirroring the fertility advantage of these groups over the control and over the 3–4 mg/mL concentrations. Unlike individual enzyme activities, T-AOC reflects the integrated capacity of the seminal environment to neutralize diverse ROS, encompassing both enzymatic and non-enzymatic antioxidant components [25]. The alignment between T-AOC and fertility in the present dataset suggests that it is the overall antioxidant buffering of the storage system, rather than the activity of any single enzyme, that most directly determines chilled semen’s ability to sustain fertilizing competence over time. This interpretation is supported by findings from other avian and mammalian systems in which TAC has been identified as a reliable predictor of post-storage sperm function and field fertility [23,24].

The antioxidant enzyme response further highlights the complexity of dose-dependent effects in this system. The 4 mg/mL group produced the highest GPx activity at 0 and 24 h and the highest CAT activity at 24 h; however, by 48 h, neither GPx nor CAT differed significantly among groups (*p* = NS and *p* = 0.2498, respectively), indicating that the enzyme-activating effect of higher ginseng concentrations was not sustained through the full storage period. More importantly, the elevated enzyme activities observed at 4 mg/mL in early storage did not translate into superior motility or fertility at any time point, demonstrating that enzyme activation alone is not a reliable surrogate for reproductive performance. This divergence points toward a hormetic dose–response relationship in which supraoptimal ginseng supplementation becomes functionally counterproductive. Comparable patterns have been reported in chilled rooster semen supplemented with MitoQ, where intermediate concentrations outperformed higher doses in both sperm quality and reproductive outcomes [26], and for cysteamine, where moderate doses improved sperm quality and fertility more effectively than higher concentrations [27]. A consistent principle has also emerged across diverse supplementation strategies in rooster semen, including phosphorus, vitamin B12, serine, and oleic acid, where intermediate rather than maximal doses yielded the strongest functional outcomes [2,12,28,29]. The present findings therefore suggest that at 4 mg/mL, ginseng was biochemically active but functionally suboptimal, and that excessive phytochemical concentrations may disrupt the seminal microenvironment in ways that impair sperm function independently of their antioxidant activity.

The biological basis for these protective effects is consistent with the established pharmacology of ginseng. Although individual ginsenosides were not isolated or characterized in the present study, ginseng extract is known to regulate ROS homeostasis, stabilize biological membranes, and protect male reproductive tissues under oxidative challenge through the actions of its principal bioactive constituents, including ginsenosides Rb1, Rg1, and Re [15,30,31,32]. In rooster semen specifically, ginseng supplementation of cryopreservation media has been shown to reduce lipid peroxidation, enhance antioxidant enzyme activity, and improve post-thaw fertility in a concentration-dependent manner [16], while dietary ginseng supplementation has been shown to improve semen quality and fertility in mature and aging Thai native roosters, indicating that ginseng can support sperm function through both systemic and direct extracellular mechanisms [17]. Outside avian systems, notoginsenoside R1 improved motility, membrane integrity, acrosomal status, and antioxidant defense during liquid storage of boar semen, further supporting the capacity of ginseng-derived compounds to exert direct cytoprotective effects within a storage medium [18]. Importantly, the present study extends this evidence to a biologically distinct context. Unlike cryopreservation, chilled storage does not expose spermatozoa to acute freeze–thaw trauma but rather to prolonged subphysiological metabolism during which oxidative damage accumulates gradually. The demonstration that ginseng remains effective under these conditions indicates that its protective value extends beyond cryoprotection to the broader stabilization of sperm function during short-term liquid storage, which is of direct practical relevance to commercial poultry AI programs.

The fertility data provide the strongest practical validation of these findings. At 0 h of storage, treatment differences in fertility were limited, with only the 2 mg/mL group significantly exceeding the 3 mg/mL group, while all other groups were intermediate. This relatively modest differentiation at 0 h is consistent with the expectation that oxidative damage requires time to accumulate to functionally significant levels, and that the antioxidant benefits of ginseng become progressively more important as storage duration increases. Accordingly, at 24 and 48 h, a clear and consistent pattern emerged: the 1 and 2 mg/mL groups maintained significantly higher fertility rates than the control group, while the 3 and 4 mg/mL groups were intermediate. This temporal progression reinforces the interpretation that ginseng-mediated antioxidant protection becomes increasingly consequential as cumulative oxidative burden rises during storage. The convergence of reduced MDA, sustained T-AOC, and improved fertility in the 1–2 mg/mL groups strongly supports the conclusion that oxidative stabilization at moderate ginseng concentrations preserved the functional integrity required for successful fertilization, consistent with previous findings linking lower oxidative burden to improved fertility in chilled chicken semen [5,23].

The optimal ginseng extract concentration identified in the present chilled storage study (1–2 mg/mL) was markedly higher than that reported to be effective in cryopreserved Pradu Hang Dam rooster semen by Ratchamak et al. [16], in which 0.25 mg/mL produced the highest post-thaw sperm quality and fertility ability. This four- to eight-fold difference in effective concentration between preservation modalities is biologically consistent and can be explained by fundamental differences in the oxidative microenvironment between cryopreservation and chilled liquid storage. During cryopreservation, sperm cells are exposed to acute, intense oxidative insult generated by phase transitions, ice crystal formation, osmotic shock, and cryoprotectant toxicity within a compressed timeframe; consequently, even low concentrations of antioxidant supplementation may be sufficient to shift the redox balance toward protection [33,34]. In contrast, chilled storage maintains spermatozoa in a metabolically active state at 5 °C for prolonged periods, during which ROS generation is continuous, cumulative, and sustained over 24–48 h; therefore, a higher antioxidant reserve in the extender is required to maintain adequate redox buffering throughout the storage period [13,35]. This interpretation is supported by the observation that T-AOC—a composite index of total antioxidant buffering capacity—was most effectively preserved at 1–2 mg/mL in the present study and aligned most closely with fertility outcomes, whereas individual enzyme activities, particularly CAT, did not differ significantly among groups at 48 h, suggesting that total antioxidant buffering rather than discrete enzyme upregulation is the principal protective mechanism under chilled storage conditions. In the cryopreservation study, by contrast, CAT was significantly elevated at 0.25–0.50 mg/mL (*p* < 0.05), indicating that acute cryogenic stress may preferentially engage H_2_O_2_-dependent antioxidant pathways [36]. These findings are consistent with recent dose-dependent evidence in rooster and mammalian semen, demonstrating that antioxidant supplementation must be tailored to the predominant sources and temporal dynamics of ROS associated with each preservation system [20,37]. Collectively, these comparisons underscore that the effective antioxidant concentration for ginseng extract is storage modality-specific and cannot be directly extrapolated between cryopreservation and liquid preservation protocols, even within the same species. They further highlight the importance of independently optimizing antioxidant supplementation for each preservation system, as both under-supplementation and over-supplementation may compromise sperm function and fertility through insufficient protection and cytotoxic or prooxidant effects, respectively [27,38].

## 5. Conclusions

The present study demonstrates that supplementation of the IGGKPh extender with Panax ginseng extract supports the preservation of chilled Leung Hang Kao rooster semen during storage at 5 °C for up to 48 h. Ginseng supplementation reduced lipid peroxidation, enhanced antioxidant enzyme activities, and maintained total antioxidant capacity in a concentration-dependent manner, with corresponding improvements in sperm viability, total motility, and progressive motility relative to the unsupplemented control. Critically, response patterns differed across parameters: sperm motility and viability were best maintained at 2–3 mg/mL, whereas total antioxidant capacity and fertility were most effectively preserved at 1–2 mg/mL. The alignment between T-AOC and fertility across storage time points indicates that overall antioxidant buffering capacity is a stronger determinant of fertilizing competence than individual enzyme activities or MDA concentration alone. Concentrations of 3–4 mg/mL, despite producing lower MDA and higher enzyme activities at certain time points, did not confer superior fertility outcomes, suggesting a hormetic dose–response relationship in which supraoptimal supplementation becomes functionally counterproductive. Based on integrated evidence from sperm quality, antioxidant status, and in vivo fertility, a ginseng extract concentration of 1–2 mg/mL is recommended as the most suitable range for practical application in the preservation of chilled rooster semen. These findings provide a scientific basis for incorporating ginseng extract as a natural antioxidant supplement in liquid semen extenders for Thai native poultry breeding programs, and support further investigation into the ginsenoside constituents responsible for the observed protective effects across diverse genetic lines and storage conditions.

## Figures and Tables

**Table 1:** Fresh semen characteristics of Leung Hang Kao roosters prior to chilled storage (n = 6 replicates).

Parameter	Mean ± SE
Volume (mL)	0.42 ± 0.10
Concentration (×10^7^ sperm/mL)	732.50 ± 76.97
Mass motility score (0–5)	3.99 ± 0.15
Total motility (%)	86.67 ± 1.29
Progressive motility (%)	79.83 ± 3.04
Viability (%)	90.55 ± 0.44

**Table 2:** Effects of ginseng extract concentration on sperm viability, total motility, and progressive motility of Leung Hang Kao rooster semen during chilled storage at 5 °C.

Parameter	Ginseng Extract (mg/mL)					SEM	*p*-Value
	0	1	2	3	4		
**Viability (%)**							
0 h	64.75 ^b^	81.41 ^a^	76.00 ^a^	73.91 ^ab^	74.33 ^ab^	1.73	0.0025
24 h	60.50 ^b^	76.16 ^a^	70.50 ^a^	76.66 ^a^	61.83 ^b^	1.87	0.0007
48 h	56.83 ^b^	72.16 ^a^	72.66 ^a^	71.83 ^a^	57.67 ^b^	1.81	<0.0001
**Total motility (%)**							
0 h	69.19 ^c^	80.95 ^a^	74.05 ^b^	75.55 ^b^	69.77 ^c^	1.40	<0.0001
24 h	64.19 ^b^	75.95 ^a^	75.95 ^a^	72.38 ^a^	64.77 ^b^	1.42	0.0001
48 h	61.23 ^c^	72.62 ^ab^	76.60 ^a^	76.95 ^a^	69.07 ^b^	1.20	<0.0001
**Progressive motility (%)**							
0 h	40.82 ^c^	52.27 ^a^	46.41 ^b^	45.85 ^b^	40.12 ^c^	1.16	<0.0001
24 h	40.85 ^bc^	48.13 ^ab^	49.45 ^a^	48.71 ^ab^	39.76 ^c^	1.17	0.0031
48 h	28.20 ^b^	35.50 ^ab^	43.87 ^a^	44.45 ^a^	34.47 ^ab^	1.74	0.0181

^a–c^ Values within the same row with different superscripts differ significantly (*p* < 0.05). Comparisons are made among ginseng extract concentrations within each storage time point. Values are presented as mean ± SEM; n = 6 replicates per treatment.

**Table 3:** Effects of ginseng extract concentration on malondialdehyde concentration and antioxidant enzyme activities of rooster semen during chilled storage at 5 °C.

Parameter	Ginseng Extract (mg/mL)					SEM	*p*-Value
	0	1	2	3	4		
**MDA (µmol/mL)**							
0 h	0.65 ^b^	0.47 ^a^	0.37 ^a^	0.38 ^a^	0.37 ^a^	0.03	<0.0001
24 h	0.66 ^b^	0.50 ^a^	0.52 ^ab^	0.51 ^ab^	0.54 ^ab^	0.02	0.0338
48 h	0.69 ^b^	0.48 ^ab^	0.47 ^ab^	0.44 ^a^	0.30 ^a^	0.04	0.0017
**T-AOC (%)**							
0 h	60.89 ^ab^	62.45 ^a^	60.00 ^ab^	57.27 ^b^	59.59 ^ab^	0.56	0.0130
24 h	60.81 ^ab^	64.34 ^a^	61.10 ^ab^	59.12 ^b^	62.89 ^ab^	0.64	0.0419
48 h	58.50 ^ab^	62.58 ^a^	62.39 ^a^	58.90 ^ab^	56.99 ^b^	0.70	0.0195
**Gpx (U/mL)**							
0 h	2.16 ^b^	3.33 ^ab^	2.91 ^ab^	2.50 ^ab^	4.58 ^a^	0.31	0.0281
24 h	3.83 ^b^	4.58 ^ab^	5.83 ^ab^	4.50 ^ab^	6.67 ^a^	0.32	0.0175
48 h	1.45 ^b^	2.92 ^a^	3.33 ^a^	2.91 ^a^	3.33 ^a^	0.23	0.0485
**CAT (U/mL)**							
0 h	8.57 ^b^	9.59 ^ab^	10.23 ^a^	9.19 ^ab^	9.37 ^ab^	0.16	0.0071
24 h	8.65 ^c^	10.23 ^bc^	10.61 ^bc^	11.09 ^b^	14.07 ^a^	0.50	0.0007
48 h	8.80	9.70	9.90	9.54	9.90	0.17	0.2498

^a–c^ Values within the same row with different superscripts differ significantly (*p* < 0.05). Comparisons are made among ginseng extract concentrations within each storage time point. Values are presented as mean ± SEM; n = 6 replicates per treatment. MDA = malondialdehyde; T-AOC = total antioxidant capacity; GPx = glutathione peroxidase; CAT = catalase.

## Data Availability

The data are available upon request from the corresponding author.
